# Factors Associated with Functional Disability and Walking Capacity in Adults with Chronic Low Back Pain: A Cross-Sectional Study

**DOI:** 10.3390/jcm15197560

**Published:** 2026-09-29

**Authors:** Moayad S. Subahi, Fahda M. Alshaikh, Hammad S. Alhasan, Ammar Fadil, Moiyad S. Aljehani, Nabeel Hamdan Alghamdi, Mansour Abdullah Alshehri

**Affiliations:** 1 Department of Medical Rehabilitation Sciences, Faculty of Applied Medical Sciences, Umm Al-Qura University, Makkah 24382, Saudi Arabia; mssubahi@uqu.edu.sa (M.S.S.); fmshiakh@uqu.edu.sa (F.M.A.); hshasan@uqu.edu.sa (H.S.A.); asfadil@uqu.edu.sa (A.F.); msjehani@uqu.edu.sa (M.S.A.); 2 Department of Physical Therapy, Faculty of Medical Rehabilitation Sciences, King Abdulaziz University, Jeddah 21589, Saudi Arabia; nhsalghamdi@kau.edu.sa

**Keywords:** chronic low back pain, CLBP, disability, walking, fear-avoidance beliefs, comorbidity

## Abstract

**Background/Objectives**: Disability in chronic low back pain (CLBP) is multifactorial, yet the independent associations of pain, fear-avoidance beliefs, and comorbidity burden with self-reported disability and performance-based walking capacity remain unclear, particularly in Saudi Arabia. This study examined factors independently associated with functional disability and walking capacity in adults with CLBP and explored whether disability was independently associated with walking capacity after accounting for demographic and clinical factors. **Methods**: This cross-sectional study included 339 adults with CLBP. Functional disability was assessed using the Oswestry Disability Index (ODI), walking capacity using the 6-Minute Walk Test (6MWT), and fear-avoidance beliefs using the physical activity (FABQ-PA) and work (FABQ-Work) subscales of the Fear-Avoidance Beliefs Questionnaire. Comorbidity burden was quantified as an unweighted count of previously diagnosed chronic conditions reported by participants and BMI-defined obesity. Multiple linear regression was used to examine factors independently associated with disability and walking capacity. The disability model included age, sex, pain intensity, FABQ-PA, FABQ-Work, and comorbidity burden. Walking capacity was examined using two models: the first included the same factors, whereas the second exploratory model additionally included ODI to examine whether disability was independently associated with 6MWT distance. **Results**: Bivariate analyses showed that pain intensity was positively correlated with FABQ-PA and FABQ-Work (r = 0.303–0.355), while ODI was positively correlated with comorbidity burden (r = 0.170). The 6MWT was negatively correlated with pain intensity, FABQ-PA, and FABQ-Work (r = −0.162 to −0.170) and more strongly with ODI (r = −0.550). In the adjusted disability model, comorbidity burden was the only factor independently associated with ODI score, with each additional comorbid condition associated with a 2.30-point higher ODI score (B = 2.296, 95% CI 0.834 to 3.759, *p* = 0.002). In the first adjusted walking-capacity model, higher pain intensity was independently associated with shorter 6MWT distance (B = −5.108, 95% CI −10.05 to −0.17, *p* = 0.044), while the other factors were not significantly associated with walking capacity. In the second adjusted walking-capacity model, greater disability was independently associated with shorter 6MWT distance (B = −3.996, 95% CI −4.655 to −3.337, *p* < 0.001). None of the other factors remained significantly associated with walking capacity in this model. **Conclusions**: Greater comorbidity burden was independently associated with greater disability in adults with CLBP, while greater disability was strongly associated with poorer walking capacity, highlighting the relevance of comorbidity burden and disability to functional limitations in CLBP.

## 1. Introduction

Low back pain (LBP) is among the leading causes of disability worldwide, affecting more than half a billion people at any given time and contributing substantially to healthcare costs and productivity loss [1]. Despite advances in clinical management, chronic low back pain (CLBP) remains a complex and heterogeneous condition with multifactorial determinants and variable functional outcomes. Persistent symptoms and associated disability are increasingly understood to reflect interactions among biological, psychological, and social factors rather than structural abnormalities alone [2].

Psychosocial factors, particularly fear-avoidance beliefs, have been consistently implicated in the transition from acute to chronic pain [1]. According to the Fear-Avoidance Model, individuals who catastrophize pain and develop fear of movement may avoid physical activity, potentially contributing to muscular deconditioning, reduced function, and persistent disability [3,4]. Empirical studies across diverse populations have supported this framework, demonstrating associations between greater fear-avoidance beliefs and higher disability, delayed recovery, and poorer rehabilitation outcomes [5,6,7]. However, the strength of these associations may vary according to pain chronicity, clinical context, and individual health characteristics. Importantly, exercise- and education-based interventions for non-specific back pain have demonstrated improvements in pain and disability, with some evidence of beneficial effects on kinesiophobia, further supporting the clinical relevance of assessing both functional disability and pain-related beliefs [8].

Alongside psychosocial factors, an emerging body of research has emphasized the potential importance of multimorbidity and the coexistence of multiple chronic conditions in pain and disability outcomes [9]. In individuals with CLBP, comorbidities such as diabetes, hypertension, obesity, and depression may contribute to functional limitations through physiological and behavioral pathways. Systemic inflammation, reduced aerobic capacity, medication effects, and reduced engagement in physical activity may collectively contribute to disability beyond the influence of pain itself [10,11,12]. Multimorbidity may also increase the complexity of self-management and potentially reduce adherence to exercise and rehabilitation programs. Despite its potential relevance, the association of comorbidity burden with disability and performance-based outcomes in CLBP has received relatively limited empirical attention.

Physical performance represents an important dimension of functional health and is frequently impaired in individuals with CLBP [12,13,14]. Objective measures such as the 6-Minute Walk Test (6MWT) provide valuable information on physical capacity and are commonly used in chronic pain research to complement self-reported outcomes [15]. The 6MWT reflects the integrated response of the cardiovascular, respiratory, and musculoskeletal systems, providing a clinically relevant measure of functional walking capacity [16]. Previous studies have demonstrated moderate correlations between 6MWT distance and disability measures such as the Oswestry Disability Index (ODI) [17], supporting the importance of evaluating both self-reported disability and performance-based function.

Recent literature also highlights the importance of considering cultural and contextual differences in pain behavior and coping strategies. In Saudi Arabia, LBP prevalence is notably high among healthcare workers, students, and community samples, with studies reporting associations between pain, sedentary lifestyle, and psychosocial distress [18,19,20]. Validated Arabic versions of both measures are available, including the ODI in a Saudi population [21] and the FABQ in Arabic-speaking patients with low back pain [22,23]. However, no prior study in the region has examined how comorbidity burden relates to psychosocial factors in relation to disability and physical performance. Taken together, existing evidence indicates that CLBP is shaped by the interplay between psychosocial mechanisms and broader health-related factors [24,25]. Nevertheless, the independent contribution of comorbidity burden to disability remains unclear. Clarifying these relationships may help explain why individuals with CLBP can experience substantially different levels of functional limitation despite comparable pain intensity.

Importantly, disability in CLBP may reflect multiple potentially interacting domains, including psychosocial factors such as fear-avoidance beliefs and broader health burden represented by comorbidity. Although the Fear-Avoidance Model has been influential in explaining persistent pain-related disability, evidence suggests that broader physiological and health-related factors may also be important determinants of functional outcomes in established CLBP [17]. Clarifying the relative contributions of these domains may improve understanding of the factors associated with functional limitations and inform clinical assessment. Moreover, examining both self-reported disability (ODI) and performance-based walking capacity (6MWT) allows complementary assessment of perceived and objectively measured function. Therefore, the present study aimed to examine the independent associations of pain intensity, fear-avoidance beliefs, and comorbidity burden with functional disability and walking capacity in adults with CLBP. A secondary exploratory aim was to determine whether self-reported disability was independently associated with walking capacity after accounting for demographic and clinical factors.

## 2. Materials and Methods

### 2.1. Study Design, Setting, and Ethics

This cross-sectional observational study was conducted in the Makkah region, Saudi Arabia, between October 2025 and June 2026. Participants were recruited from physiotherapy outpatient departments, primary healthcare centers, and community settings, with additional recruitment through online advertisements distributed via professional physiotherapy networks and social media platforms. Online advertisements and social media were used solely as recruitment channels. Individuals recruited through these channels subsequently attended an in-person study assessment, during which all questionnaires, anthropometric measurements, and physical performance testing, including the supervised 6MWT, were completed using the same standardized procedures as for participants recruited through the other channels. Recruitment source was not recorded at the individual-participant level; therefore, the number of participants recruited through each channel could not be determined retrospectively. The study was conducted and reported following the Strengthening the Reporting of Observational Studies in Epidemiology (STROBE) guidelines (Supplementary File S1). The study protocol was approved by the Local Committee for Biological and Medical Ethics at Umm Al-Qura University (Approval No. HAPO-02-K-012-2025-10-2939), and all procedures were conducted in accordance with the Declaration of Helsinki. All participants provided informed consent before participation. All data were anonymized, coded, and stored in a secure database accessible only to the research team.

### 2.2. Participants

Adults aged ≥18 years with CLBP were eligible for inclusion. Chronicity was determined based on self-reported pain duration. Participants reporting pain lasting “from 3 to less than 6 months” or “more than 6 months” were classified as having CLBP (≥12 weeks). Exclusion criteria were: (1) participant-reported history of physician-diagnosed specific spinal pathology (e.g., fracture, infection, inflammatory disease, or malignancy); (2) spinal surgery within the previous 12 months; and (3) neurological, cognitive, or psychiatric conditions that could impair accurate self-reporting or safe participation in physical testing. A history of depression was not itself an exclusion criterion unless the condition was of sufficient severity to compromise accurate questionnaire completion or safe participation in physical testing; therefore, participants with a previous diagnosis of depression who were able to complete the study procedures were retained and depression was counted as one component of the comorbidity burden. Accordingly, the analytical sample represented adults with non-specific CLBP, defined as low back pain persisting for ≥12 weeks without a reported history of physician-diagnosed specific spinal pathology.

### 2.3. Sample Size

The required sample size was estimated using G*Power 3.1 for a fixed-model multiple linear regression analysis (R^2^ deviation from zero), assuming a medium effect size (f^2^ = 0.15), α = 0.05, 90% statistical power, and six predictors. The corrected minimum required sample size was 123 participants. A total of 339 participants were included in the final analysis, substantially exceeding this requirement.

### 2.4. Measures

#### 2.4.1. Demographic and Clinical Variables

Participants reported their age, sex, education level, employment status, duration of low back pain, and pain intensity using a 0–10 numeric rating scale. Height and body mass were obtained during the study assessment, and body mass index (BMI) was calculated as body mass in kilograms divided by height in meters squared (kg/m^2^). The comorbidity checklist was developed specifically for this study and was not adapted from an existing validated comorbidity instrument. Comorbidity burden was operationalized as an unweighted count of six participant-reported previously diagnosed chronic conditions and BMI-defined obesity. Participants indicated whether they had previously received a clinical diagnosis of hypertension, diabetes, heart disease, osteoarthritis, asthma/chronic obstructive pulmonary disease (COPD), or depression. These conditions were reported by participants based on previous clinical diagnoses and were not independently diagnosed or verified by the research team. Obesity was determined separately by the research team from BMI, calculated from height and weight, with BMI ≥ 30 kg/m^2^ classified as obesity. Each of the seven conditions contributed one point to the comorbidity burden score, yielding a theoretical range of 0–7, with higher scores indicating greater comorbidity burden. The study-specific recruitment and clinical information checklist, including the complete comorbidity checklist, is provided as Supplementary File S2.

#### 2.4.2. Fear-Avoidance Beliefs

Fear-avoidance beliefs were assessed using the validated Arabic version of the FABQ. The FABQ comprises two subscales: Physical Activity (FABQ-PA; 4 items) and Work (FABQ-Work; 7 items). Each item is rated on a 7-point Likert scale ranging from 0 (“completely disagree”) to 6 (“completely agree”), with higher subscale scores indicating stronger fear-avoidance beliefs. The administered questionnaire retained the standard 0–6 response options. In the present analytical sample, however, the observed responses to the scored FABQ items ranged from 1 to 5; no response of 0 or 6 was observed. The two subscale scores were analyzed separately.

#### 2.4.3. Functional Disability

Functional disability was assessed using the ODI [26]. The ODI consists of 10 items assessing pain-related limitations in daily activities. Each item is scored from 0 to 5, yielding a raw total score of 0–50, which is converted to a percentage (0–100%), with higher scores indicating greater disability. The Arabic version of the ODI has demonstrated robust reliability and validity in Saudi populations [21].

#### 2.4.4. Physical Performance

Participants completed the self-reported questionnaires before undertaking the physical performance assessment. Assessors administering the 6MWT were not formally blinded to questionnaire responses; however, the 6MWT was administered using the same standardized protocol for all participants. Performance-based walking capacity was assessed using the 6MWT, following American College of Sports Medicine guidance [16]. The test was performed indoors on a flat, standardized 30 m walking course. Participants were instructed to walk as far as possible for six minutes, and standardized verbal encouragement was provided throughout the test. Participants were permitted to slow down or rest, if necessary, with the test time continuing during rest periods, and those who routinely used a walking aid were permitted to use it during the test. Heart rate and blood pressure were assessed before and after the test for safety. The test was terminated if a participant developed chest pain, severe dyspnea, dizziness, loss of balance, or other symptoms indicating that continuation was unsafe. Any safety-related interruption requiring cessation of the test was recorded. The total distance walked during the six-minute period was recorded in meters and used as the performance outcome. The same standardized procedure was applied to all participants. All assessors received standardized instruction in the 6MWT protocol before data collection to ensure consistent test administration.

### 2.5. Statistical Analysis

Statistical analyses comprise descriptive statistics, bivariate correlation analyses, and multivariable regression modeling. Continuous variables were screened for plausibility, missing values, and outliers. Pearson correlation coefficients were calculated to examine bivariate associations among continuous variables. Because comorbidity burden was represented as a count variable, Spearman rank correlations were additionally performed as a sensitivity analysis for its associations with the study variables. Moreover, to account for multiple testing across the 21 pairwise correlations, *p*-values were adjusted using the Benjamini–Hochberg false discovery rate procedure.

The primary models were specified a priori as parsimonious, hypothesis-driven models focused on pain intensity, fear-avoidance beliefs, and comorbidity burden, with age and sex included as demographic covariates. Multiple linear regression analyses were conducted for the two primary outcomes. First, functional disability (ODI score) was modeled as the dependent variable to examine factors independently associated with disability. The independent variables were age, sex, pain intensity, fear-avoidance beliefs (FABQ-PA and FABQ-Work), and comorbidity burden.

Second, performance-based walking capacity (6MWT distance) was examined using two sequential models. Model 1 included age, sex, pain intensity, FABQ-PA, FABQ-Work, and comorbidity burden to examine their independent associations with walking capacity. Model 2 additionally included ODI score to examine whether self-reported disability was independently associated with walking capacity after accounting for the demographic and clinical variables included in Model 1. Because ODI was not included as a predictor in the initial sample size estimation, Model 2 was considered exploratory.

Regression assumptions were evaluated using residual-versus-predicted and Q–Q plots, Durbin–Watson statistics, variance inflation factors (VIFs), standardized residuals, and Cook’s distance. No major violations of linearity, homoscedasticity, residual normality, independence, or influential-case assumptions were identified. Durbin–Watson statistics were 1.846 for the ODI model and 1.970 for the final 6MWT model; maximum Cook’s distances were well below 1. VIF values ranged from approximately 1.00 to 3.04, with the highest values observed for the correlated FABQ subscales.

All predictors were entered simultaneously within each model. Regression coefficients, 95% confidence intervals (CIs), and model fit indices were reported. To examine the sensitivity of the primary findings to additional potentially relevant covariates available in the dataset, exploratory sensitivity analyses were conducted for both ODI and 6MWT. These expanded models included the predictors from the primary models together with BMI, pain duration, Pain Catastrophizing Scale (PCS), Pittsburgh Sleep Quality Index (PSQI), and occupational status. These variables were selected because they represent additional anthropometric, clinical, psychological, sleep-related, and occupational factors that may be associated with disability or walking capacity in CLBP. The expanded models were considered exploratory and were used to assess whether the associations observed in the primary models were materially altered after broader covariate adjustment.

Given the strong correlation between the FABQ-PA and FABQ-Work subscales, both variables were retained in the primary regression models to preserve their domain-specific information. A post hoc total FABQ score was not constructed because the Physical Activity and Work subscales represent distinct fear-avoidance domains and the prespecified analyses were based on these domain-specific scores. VIFs were examined to assess multicollinearity, with values around 3 interpreted cautiously in conjunction with the observed correlation between the FABQ subscales. To determine whether simultaneous inclusion of the correlated FABQ subscales influenced their individual regression estimates, sensitivity analyses were conducted by repeating the ODI and 6MWT models with FABQ-PA and FABQ-Work entered separately while retaining the remaining covariates. For 6MWT, these separate-subscale analyses were performed both without and with ODI included as an additional predictor. Because the ODI includes items assessing walking and standing, an additional sensitivity analysis was performed to assess potential content overlap with the 6MWT. A modified ODI score was calculated after excluding the Walking and Standing items and rescaling the remaining eight items to a 0–100 scale. The exploratory 6MWT Model 2 was then repeated using this modified ODI score in place of the original ODI. To further evaluate the measurement characteristics of the ODI and FABQ in the present sample, internal consistency was assessed using Cronbach’s alpha for the ODI, FABQ-PA, and FABQ-Work. Score distributions were additionally summarized using the mean, standard deviation, median, interquartile range, observed range, skewness, and kurtosis. No missing data were identified; therefore, all analyses were conducted using the full dataset. Statistical analyses were performed using JASP (version 0.95.3), with statistical significance set at *p* < 0.05.

## 3. Results

### 3.1. Sample Characteristics

**Figure 1:**
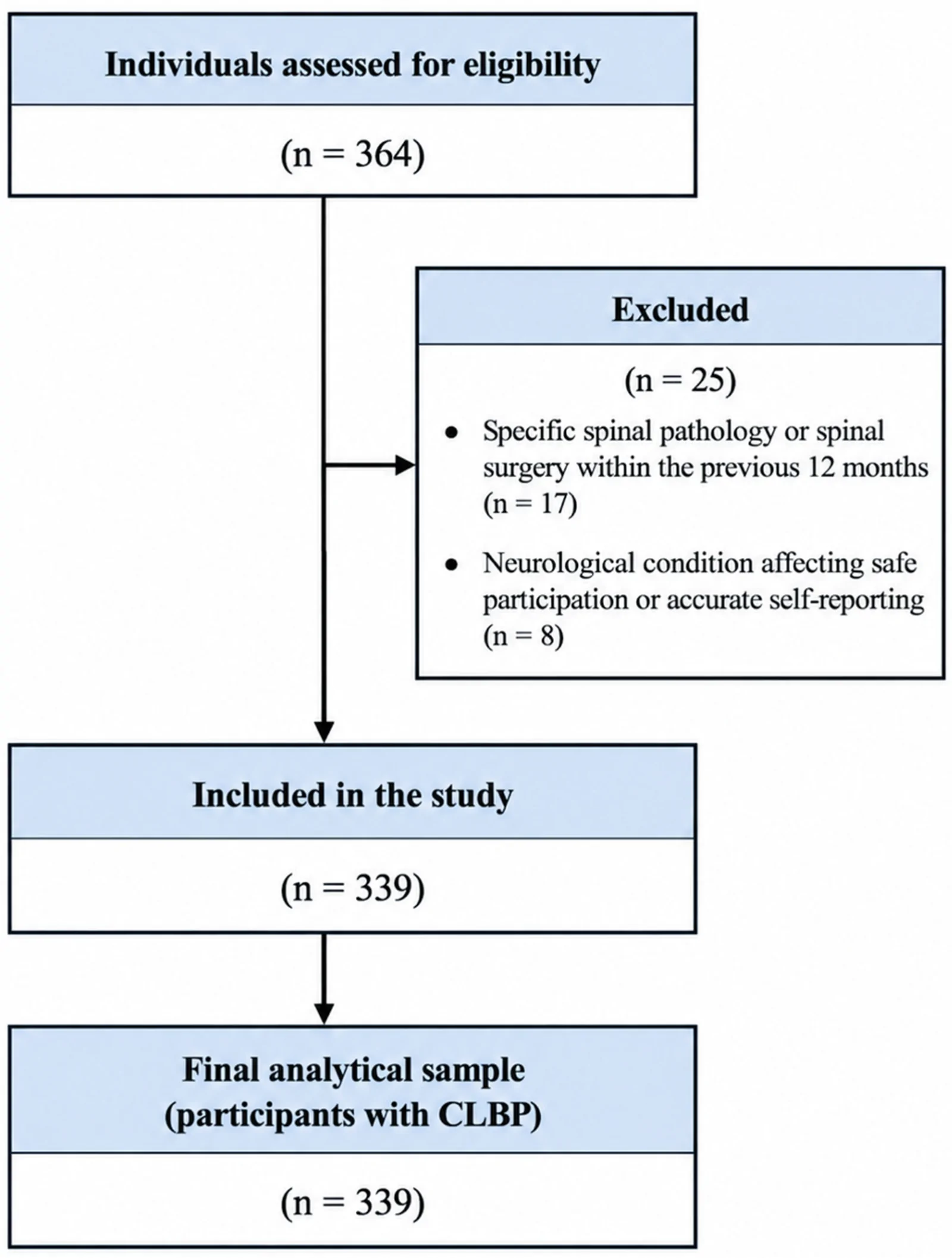
Study flow diagram.

A total of 364 individuals were assessed for eligibility. Of these, 25 were excluded because of specific spinal pathology or spinal surgery within the previous 12 months (n = 17) or a neurological condition affecting safe participation or accurate self-reporting (n = 8), resulting in a final analytical sample of 339 participants with CLBP (Figure 1). The sample was predominantly female (69.3%), with a mean age of 48.31 ± 10.05 years. Mean height was 1.66 ± 0.10 m, mean weight was 69.74 ± 14.04 kg, and mean BMI was 25.35 ± 4.11 kg/m^2^. Mean pain intensity was 5.95 ± 2.37, and the mean ODI score was 43.88 ± 14.22. The mean 6MWT distance was 403.12 ± 104.49 m. No participant required premature termination of the 6MWT because of safety-related symptoms, and no safety-related adverse event requiring test cessation was recorded. The mean comorbidity burden was 2.08 ± 1.04 conditions (observed range, 0–5). Participant characteristics and the frequencies of individual comorbid conditions are presented in Table 1.

Inspection of the item-level FABQ data confirmed that the administered questionnaire used the standard 0–6 response scale, although observed responses to all scored items ranged from 1 to 5 in the present sample. Consequently, the observed FABQ-PA and FABQ-Work total scores ranged from 4 to 20 and 7 to 35, respectively. Internal consistency was high for both FABQ-PA (α = 0.851) and FABQ-Work (α = 0.908). Detailed distributional characteristics and item-level response frequencies are presented in Supplementary Table S1.

### 3.2. Bivariate Associations

Pearson correlation analyses showed that pain intensity was positively correlated with both FABQ-PA (r = 0.355, *p* < 0.001) and FABQ-Work (r = 0.303, *p* < 0.001). The two FABQ subscales were strongly positively correlated (r = 0.807, *p* < 0.001). Functional disability, assessed using the ODI, showed a weak positive correlation with comorbidity burden (r = 0.170, BH-adjusted *p* = 0.006). In contrast, ODI scores were not significantly correlated with pain intensity, FABQ-PA, or FABQ-Work.

Performance-based walking capacity, assessed using the 6MWT, was moderately negatively correlated with ODI score (r = −0.550, *p* < 0.001), with greater self-reported disability corresponding to shorter walking distance. Weak negative correlations were also observed between 6MWT distance and pain intensity (r = −0.167, *p* = 0.002), FABQ-PA (r = −0.162, *p* = 0.003), and FABQ-Work (r = −0.170, *p* = 0.002). The correlation between 6MWT distance and comorbidity burden was not statistically significant. Bivariate correlations among the study variables are presented in Table 2.

In a sensitivity analysis using Spearman rank correlations, the pattern of associations involving comorbidity burden was unchanged (Supplementary Table S2). Comorbidity burden remained positively associated with ODI (ρ = 0.148, *p* = 0.006), whereas its association with 6MWT distance remained non-significant (ρ = −0.072, *p* = 0.187). Associations with age, pain intensity, FABQ-PA, and FABQ-Work also remained non-significant. All correlations that were statistically significant in the unadjusted analysis remained significant after Benjamini–Hochberg false discovery rate correction for the 21 pairwise comparisons.

### 3.3. Multivariable Analysis of Functional Disability

The overall model was statistically significant (*p* = 0.043), although it explained a small proportion of the variance in ODI scores (R^2^ = 0.038; adjusted R^2^ = 0.021). Comorbidity burden was the only factor independently associated with functional disability. Each additional comorbid condition was associated with a 2.30-point higher ODI score (B = 2.296, 95% CI 0.834 to 3.759, *p* = 0.002). Pain intensity, FABQ-PA, FABQ-Work, age, and sex were not independently associated with ODI score after adjustment. Multicollinearity diagnostics indicated acceptable levels, VIF values ranged from approximately 1.00 to 3.04, with the highest values observed for the correlated FABQ subscales. In separate-subscale sensitivity analyses, neither FABQ-PA (B = −0.037, 95% CI −0.372 to 0.298, β = −0.012, *p* = 0.830) nor FABQ-Work (B = −0.043, 95% CI −0.233 to 0.147, β = −0.025, *p* = 0.656) was independently associated with ODI. Comorbidity burden remained independently associated with ODI in both separate-subscale models (both *p* = 0.002; Supplementary Table S3). Full multivariable analysis results are presented in Table 3.

#### Sensitivity Analysis with Additional Covariates

In an exploratory sensitivity analysis, the ODI regression model was expanded to additionally include BMI, pain duration, pain catastrophizing, sleep quality, and occupational status. The expanded model was statistically significant, F(13,325) = 2.70, *p* = 0.001, and explained 9.7% of the variance in ODI scores (R^2^ = 0.097; adjusted R^2^ = 0.061), compared with 3.8% in the primary model. Importantly, the association between comorbidity burden and disability remained materially unchanged (B = 2.345, 95% CI 0.904 to 3.787, β = 0.172, *p* = 0.002). BMI, pain duration, PCS, PSQI, age, sex, pain intensity, and both FABQ subscales were not independently associated with ODI in the expanded model. Occupational status showed an association for the student category relative to the reference category (B = −7.753, 95% CI −12.172 to −3.335, *p* < 0.001). Full results of the expanded ODI model are presented in Supplementary Table S4.

### 3.4. Multivariable Analysis of Walking Capacity (6MWT)

The first multiple linear regression model examining walking capacity was statistically significant (*p* = 0.005) and explained 5.5% of the variance in 6MWT distance (R^2^ = 0.055; adjusted R^2^ = 0.038). Higher pain intensity was independently associated with shorter 6MWT distance (B = −5.108, 95% CI −10.05 to −0.17, *p* = 0.044), whereas the remaining variables were not independently associated with walking capacity.

In the exploratory second model, which additionally included ODI, the explained variance increased to 33.9% (R^2^ = 0.339; adjusted R^2^ = 0.325; *p* < 0.001). Greater disability was independently associated with shorter 6MWT distance (B = −3.996, 95% CI −4.655 to −3.337, *p* < 0.001). A sensitivity analysis excluding the Walking and Standing items from the ODI yielded a similar association (B = −3.591, 95% CI −4.222 to −2.960, *p* < 0.001; R^2^ = 0.314), indicating that the association persisted after removal of the ODI items most directly related to walking performance. Full results are presented in Table 4 and Supplementary Table S5.

#### Sensitivity Analyses for the FABQ Subscales

Given the strong correlation between FABQ-PA and FABQ-Work, additional models were examined with each subscale entered separately. In models without ODI, higher FABQ-PA was independently associated with shorter 6MWT distance (B = −2.750, 95% CI −5.196 to −0.304, β = −0.127, *p* = 0.028), as was higher FABQ-Work (B = −1.785, 95% CI −3.170 to −0.400, β = −0.142, *p* = 0.012). These associations persisted after ODI was additionally included in the respective models (FABQ-PA: B = −2.895, 95% CI −4.949 to −0.842, β = −0.134, *p* = 0.006; FABQ-Work: B = −1.957, 95% CI −3.118 to −0.797, β = −0.156, *p* = 0.001). Full results of the separate-subscale models are presented in Supplementary Table S3.

The expanded sensitivity model for 6MWT was statistically significant, F(13,325) = 11.27, *p* < 0.001, and explained 31.1% of the variance in walking distance (R^2^ = 0.311; adjusted R^2^ = 0.283). Higher pain intensity (B = −4.773, 95% CI −9.108 to −0.438, *p* = 0.031) and greater comorbidity burden (B = −11.113, 95% CI −20.371 to −1.855, *p* = 0.019) were independently associated with shorter walking distance. FABQ-PA and FABQ-Work were not independently associated with 6MWT distance in this model. Occupational status was also associated with walking distance for the non-working, private-sector, and student categories relative to the reference category. Full results are presented in Supplementary Table S4.

## 4. Discussion

This study examined the independent associations of pain intensity, fear-avoidance beliefs, and comorbidity burden with functional disability and performance-based walking capacity in adults with CLBP, and further explored whether self-reported disability was independently associated with walking capacity. The findings showed distinct patterns across the disability and walking-capacity outcomes and across model specifications. Comorbidity burden was the only factor independently associated with functional disability, whereas greater disability was strongly associated with shorter 6MWT distance. Fear-avoidance beliefs were not independently associated with functional disability, whereas their associations with walking capacity varied according to model specification: neither FABQ subscale was significant when both were entered simultaneously, but each showed a modest inverse association with 6MWT distance when modeled separately.

A key finding was that comorbidity burden, rather than pain intensity or fear-avoidance beliefs, was the only factor independently associated with functional disability. Each additional comorbid condition was associated with a higher ODI score, although the magnitude of this association was modest and the overall disability model explained only a small proportion of the variance. Nevertheless, this finding highlights the potential importance of broader health burden in understanding functional limitations among adults with CLBP and is consistent with increasing recognition of multimorbidity as an important component of pain-related disability [27,28,29].

The magnitude of this association should be interpreted cautiously in relation to published thresholds for meaningful ODI change. A 10-point change on the 0–100 ODI has been proposed as a reasonable minimal important change for individuals with low back pain, although estimates vary according to population and methodology [30]. The 2.30-point adjusted difference associated with each additional comorbid condition is smaller than this commonly cited change threshold. However, minimal important change values are intended primarily for interpreting within-person longitudinal change, whereas the present estimate represents a cross-sectional between-person association. Accordingly, the 2.30-point coefficient should not be interpreted directly as either clinically important or clinically unimportant on the basis of an MIC threshold.

The exploratory sensitivity analyses provided additional context for these findings. After broader adjustment for BMI, pain duration, pain catastrophizing, sleep quality, and occupational status, the association between comorbidity burden and ODI remained similar in magnitude to that observed in the primary model. Although the expanded model explained a greater proportion of ODI variance than the primary model, its explanatory capacity remained modest, indicating that substantial variability in disability was attributable to factors not captured by the present models. Thus, the association between comorbidity burden and disability should be interpreted as modest rather than as evidence that comorbidity is a dominant determinant of disability.

The absence of an independent association between fear-avoidance beliefs and functional disability contrasts with traditional biopsychosocial frameworks that emphasize fear avoidance as an important mechanism underlying chronic pain-related disability [7,31]. However, these findings do not imply that fear-avoidance beliefs are unimportant in CLBP. Rather, they suggest that their independent association with disability may be limited when considered simultaneously with pain intensity, comorbidity burden, and demographic characteristics in this population. The strong correlation between FABQ-PA and FABQ-Work (r = 0.807) indicated substantial overlap between the two fear-avoidance domains. Although VIF values remained below conventional thresholds, the separate-subscale sensitivity analyses provided important additional context. Neither FABQ domain was associated with ODI when modeled separately. In contrast, both FABQ-PA and FABQ-Work were modestly associated with shorter 6MWT distance when entered in separate models, and these associations persisted after additional adjustment for ODI. This pattern is compatible with substantial shared variance between the two FABQ domains influencing their individual coefficients when both were entered simultaneously. Accordingly, the nonsignificant FABQ coefficients in the primary 6MWT models should not be interpreted as evidence of an absence of association between fear-avoidance beliefs and walking capacity. These findings indicate that the relationship between fear-avoidance beliefs and walking performance is sensitive to model specification and should therefore be interpreted cautiously.

The analysis of performance-based walking capacity provided additional insight. In Model 1, greater pain intensity was independently associated with shorter 6MWT distance, although the magnitude of the association was small, while comorbidity burden approached but did not reach statistical significance. When ODI score was added in the exploratory Model 2, the proportion of explained variance increased substantially from 5.5% to 33.9%, and greater disability was strongly associated with shorter walking distance. Moreover, given that ODI and 6MWT assess related dimensions of functional status, the strong association between them should not be interpreted as evidence of a causal or mechanistic relationship. Pain intensity, comorbidity burden, fear-avoidance beliefs, age, and sex were no longer independently associated with 6MWT distance in this model. This pattern indicates that self-reported disability accounted for substantially more variation in walking capacity than the other measured factors when they were considered simultaneously [29]. However, because the analysis was cross-sectional, these findings should not be interpreted as demonstrating that disability mediates the effects of pain or comorbidity on walking performance. Age and sex were not independently associated with 6MWT distance in the present multivariable models. This finding should be interpreted cautiously and should not be taken to indicate that age and sex are unimportant factors associated with walking capacity in general. The relatively restricted age distribution of the sample, the predominance of female participants, and adjustment for other clinical and functional variables may have reduced the independent contribution of age and sex in these models. In addition, 6MWT performance reflects multiple interacting physiological, clinical, and behavioral factors, so the effects of demographic characteristics may differ across populations and model specifications.

For 6MWT performance, broader covariate adjustment produced a different pattern. The expanded sensitivity model explained substantially more variance than the primary model without ODI, largely reflecting the contribution of occupational status and other covariate adjustment. Notably, the FABQ-PA coefficient changed sign after occupational status was introduced into the expanded model, while remaining statistically nonsignificant. This pattern, together with the substantial shared variance between FABQ-PA and FABQ-Work, indicates sensitivity of the FABQ coefficients to covariate specification and argues against interpreting their direction or magnitude as stable across models. Pain intensity remained independently associated with walking distance, while comorbidity burden, which showed only a borderline association in the primary model, was independently associated with shorter walking distance in the expanded model. These exploratory findings suggest that the relationship between comorbidity and walking performance may depend partly on the broader covariate structure considered and should therefore be interpreted cautiously. Potential item overlap between the ODI and 6MWT was also considered because the ODI contains specific Walking and Standing items. When these two items were removed and the modified ODI was entered into the sensitivity model, its association with 6MWT distance remained strong, although the explained variance decreased from 33.9% to 31.4%. This suggests that direct content overlap contributes only partly to the observed association. More broadly, self-reported and performance-based measures of function capture related but distinct aspects of disability in CLBP, and previous studies have similarly reported only moderate correspondence between perceived disability and objectively measured functional performance [32].

Taken together, the findings indicate that broader health burden and self-reported functional limitation may be particularly relevant to functional outcomes in this CLBP population. Comorbidity burden was independently associated with disability, whereas disability showed a strong independent association with performance-based walking capacity. From a clinical perspective, these findings suggest that assessment of functional limitations in adults with CLBP may benefit from considering multimorbidity and broader health status alongside pain and fear-avoidance beliefs. A broader assessment that incorporates multimorbidity, pain, self-reported disability, and performance-based functional capacity may therefore provide a more comprehensive characterization of functional status [12,33].

The findings also emphasize the distinction between self-reported disability and performance-based function. Although ODI scores were strongly associated with 6MWT distance, these measures assess different aspects of functional health. The ODI reflects perceived limitations across daily activities, whereas the 6MWT provides a performance-based measure of walking capacity. The substantial association between these outcomes supports their relatedness but does not indicate that they are interchangeable. Accordingly, incorporating both self-reported and performance-based measures may provide a more comprehensive characterization of functional status in individuals with CLBP [27].

Previous research has consistently identified fear-avoidance beliefs as an important correlate of disability in chronic pain populations [5,31]. However, differences in study populations, disease chronicity, covariate selection, and analytical approaches may partly explain why such associations were not independently observed in the present study. The eligibility criteria may also have contributed to these findings. Although a history of depression was not itself an exclusion criterion, individuals with psychiatric conditions severe enough to impair accurate self-reporting or safe participation in physical testing were excluded. This may have reduced the representation of participants with more severe psychological burden and potentially attenuated the observed associations between fear-avoidance beliefs and functional outcomes. Accordingly, the absence of independent associations between fear-avoidance beliefs and disability should be interpreted in the context of these eligibility criteria. In particular, studies that do not account for broader health burden may estimate the association of psychosocial factors differently. The present findings therefore raise the possibility that comorbidity burden may represent an important, yet comparatively underexamined, correlate of disability in CLBP [27,28]. At the same time, the low explanatory power of the disability model reinforces the multifactorial nature of CLBP. The variables included in the model explained only 3.8% of the variance in ODI scores, indicating that other relevant biological, psychological, behavioral, social, or environmental factors were not captured. Potential contributors may include physical activity, sleep, pain duration, psychological distress, socioeconomic circumstances, occupational demands, and other dimensions of health status.

Several limitations should be acknowledged. First, the cross-sectional design precludes causal inference and does not establish the temporal direction of the observed associations. The use of online recruitment may also have introduced selection bias and may limit the representativeness of the study sample. Second, comorbidity burden was quantified using an unweighted count of self-reported chronic conditions. These participant-reported diagnoses were not independently verified against medical records. Although this approach is straightforward and clinically interpretable, it does not account for differences in disease severity, duration, treatment burden, or the relative functional impact of individual conditions. More detailed measures of multimorbidity may therefore provide additional insight. Third, physical activity and broader psychological distress were not directly assessed and therefore could not be examined as potential factors associated with disability or walking capacity. Fourth, potential content overlap between the ODI and 6MWT should be acknowledged because the ODI includes items related to walking and standing. However, the sensitivity analysis excluding these items showed that the association with 6MWT distance persisted. Finally, although no major violations of regression assumptions were identified, the disability model explained only a small proportion of the variance in ODI scores. This indicates that important factors associated with disability were not captured in the present analysis. In addition, the ODI demonstrated modest internal consistency in the present sample (Cronbach’s α = 0.631). Although this warrants caution when interpreting ODI-based associations, the modest reliability alone does not adequately explain the weak correlations observed between ODI and pain intensity or fear-avoidance beliefs. These unexpectedly weak associations may reflect sample-specific characteristics, restricted covariance among the measured constructs, or other unmeasured factors and should therefore be interpreted cautiously. The predominance of female participants, relatively limited age distribution, and recruitment confined to the Makkah region may limit generalizability to more demographically and geographically diverse CLBP populations. These sample characteristics may also have reduced the ability to detect independent associations of age and sex with 6MWT performance. Potential heterogeneity according to sex, age, or comorbidity burden was not examined using prespecified subgroup analyses and should be evaluated in adequately powered future studies.

Future research should use longitudinal designs to clarify the temporal relationships among comorbidity burden, self-reported disability, pain, and performance-based walking capacity. Future studies should also prospectively examine effect modification by sex, age, and comorbidity burden using adequately powered subgroup or interaction analyses. Clinically meaningful thresholds for disability or walking impairment should be derived and externally validated against appropriate clinical reference outcomes rather than generated post hoc from the present cross-sectional dataset. Such studies could determine whether changes in comorbidity burden, disability, or physical capacity precede changes in other functional outcomes and thereby provide stronger evidence regarding potential pathways. Future work should also examine additional factors associated with disability in CLBP, including physical activity, psychological distress, sleep, socioeconomic characteristics, occupational demands, and environmental influences. More comprehensive measures of multimorbidity that incorporate disease severity and functional impact may further clarify the contribution of broader health burden to disability in CLBP.

## 5. Conclusions

In adults with chronic low back pain, comorbidity burden was the only factor independently associated with functional disability, whereas greater self-reported disability was strongly associated with poorer performance-based walking capacity. These findings highlight the potential relevance of multimorbidity and overall functional limitation when evaluating functional outcomes in CLBP, while also indicating that the factors associated with disability extend beyond those examined in the present study.

## Figures and Tables

**Table 1:** Participant Characteristics.

Variable		Value
Sex	Female	235 (69.3%)
	Male	104 (30.7%)
Age (years)	-	48.31 ± 10.05
Pain intensity (0–10)	-	5.95 ± 2.37
FABQ-Physical Activity	-	12.11 ± 4.84
FABQ-Work	-	21.25 ± 8.34
ODI (0–100)	-	43.88 ± 14.22
6MWT (meters)	-	403.12 ± 104.49
Comorbidity burden	-	2.08 ± 1.04
	Hypertension	158 (46.6%)
	Diabetes	141 (41.6%)
	Heart Disease	109 (32.2%)
	Asthma/COPD	85 (25.1%)
	Osteoarthritis	70 (20.6%)
	Obesity	28 (8.3%)
	Depression	114 (33.6%)
Height (m)	-	1.66 ± 0.10
Weight (kg)	-	69.74 ± 14.04
BMI (kg/m^2^)	-	25.35 ± 4.11
PCS	-	20.53 ± 9.74
PSQI	-	8.52 ± 3.39

Abbreviations: FABQ, Fear-Avoidance Beliefs Questionnaire; ODI, Oswestry Disability Index; 6MWT, 6-Minute Walk Test; BMI, Body Mass Index; PCS, Pain Catastrophizing Scale; PSQI, Pittsburgh Sleep Quality Index. Values are presented as mean ± standard deviation or n (%), as appropriate.

**Table 2:** Pearson correlations among the main variables.

Variable	Pain	FABQ-PA	FABQ-Work	ODI	6MWT	Age	Comorbidity
Pain	—						
FABQ-PA	0.355 ***	—					
FABQ-Work	0.303 ***	0.807 ***	—				
ODI	0.090	0.008	−0.010	—			
6MWT	−0.167 **	−0.162 **	−0.170 **	−0.550 ***	—		
Age	0.019	−0.007	0.010	0.030	0.002	—	
Comorbidity	0.030	−0.078	−0.080	0.170 **	−0.095	−0.007	—

Abbreviations: FABQ-PA, Fear-Avoidance Beliefs Questionnaire–Physical Activity subscale; FABQ-Work, Fear-Avoidance Beliefs Questionnaire–Work subscale; ODI, Oswestry Disability Index; 6MWT, 6-Minute Walk Test. Values are Pearson correlation coefficients (r). All tests were two-tailed. *p*-values for the 21 pairwise correlations were adjusted for multiple comparisons using the Benjamini–Hochberg false discovery rate procedure. ** *p* < 0.01, *** *p* < 0.001 after adjustment.

**Table 3:** Multiple linear regression analysis of functional disability (ODI).

Variable	Coefficient	95% CI	*p* Value
Age	0.042	−0.108 to 0.192	0.583
Pain intensity	0.536	−0.146 to 1.217	0.123
Sex (male vs. female)	−0.648	−3.955 to 2.660	0.700
FABQ-PA	0.061	−0.482 to 0.603	0.826
FABQ-Work	−0.070	−0.378 to 0.238	0.654
Comorbidity	2.296	0.834 to 3.759	0.002

ODI, Oswestry Disability Index; CI, confidence interval; FABQ, Fear-Avoidance Beliefs Questionnaire; PA, physical activity. Model R^2^ = 0.038; adjusted R^2^ = 0.021; *p* = 0.043.

**Table 4:** Multiple linear regression of factors associated with walking capacity (6MWT).

Variable	Model 1			Model 2		
	Coefficient	95% CI	*p* Value	Coefficient	95% CI	*p* Value
Age	0.050	−1.05 to 1.15	0.928	0.217	−0.698 to 1.132	0.641
Pain intensity	−5.108	−10.05 to −0.17	0.044	−2.967	−7.141 to 1.207	0.163
Sex (male vs. female)	2.845	−21.09 to 26.78	0.816	0.258	−19.930 to 20.445	0.980
FABQ-PA	−0.726	−4.68 to 3.23	0.718	−0.484	−3.796 to 2.828	0.774
FABQ-Work	−1.461	−3.73 to 0.81	0.201	−1.741	−3.622 to 0.139	0.069
Comorbidity	−10.542	−21.19 to 0.11	0.052	−1.365	−10.415 to 7.685	0.767
ODI	—	—	—	−3.996	−4.655 to −3.337	<0.001

Abbreviations: 6MWT = 6-Minute Walk Test; CI, confidence interval; FABQ, Fear-Avoidance Beliefs Questionnaire; PA, physical activity; ODI, Oswestry Disability Index. Model 1: R^2^ = 0.055, adjusted R^2^ = 0.038, *p* = 0.005. Model 2: R^2^ = 0.339, adjusted R^2^ = 0.325, *p* < 0.001.

## Data Availability

The original contributions presented in this study are included in the article; further inquiries can be directed to the corresponding author.

## Supplementary Materials

The following supporting information can be downloaded at https://www.mdpi.com/article/10.3390/jcm15197560/s1. Supplementary File S1: STROBE Statement—Checklist of items that should be included in reports of cross-sectional studies; Supplementary File S2: Study recruitment and clinical information checklist; Supplementary File S3: Supplementary Table S1: Distributional characteristics, internal consistency, and item-level response frequencies of the study measures; Supplementary Table S2: Spearman rank correlations between comorbidity burden and study variables; Supplementary Table S3: Sensitivity analyses examining FABQ subscales separately in regression models of disability and walking capacity; Supplementary Table S4: Expanded sensitivity regression models for disability (ODI) and walking capacity (6MWT); Supplementary Table S5: Sensitivity analysis of walking capacity using a modified ODI excluding the Walking and Standing items.

## Abbreviations

The following abbreviations are used in this manuscript:

LBP	Low back pain
CLBP	Chronic low back pain
6MWT	6-Minute Walk Test
ODI	Oswestry Disability Index
FABQ	Fear-Avoidance Beliefs Questionnaire
PA	Physical Activity
STROBE	Strengthening the Reporting of Observational Studies in Epidemiology
BMI	body mass index
CIs	Confidence intervals
VIFs	Variance inflation factors
PCS	Pain Catastrophizing Scale
PSQI	Pittsburgh Sleep Quality Index
