# Supplementary material for: Factors Associated with Functional Disability and Walking Capacity in Adults with Chronic Low Back Pain: A Cross-Sectional Study
Source: J Clin Med. 2026 Sep 29;15(19):7560. doi: 10.3390/jcm15197560 (PMC13666209; doi:10.3390/jcm15197560)
Supplement: Supplementary file 1 [file jcm-15-07560-s001.zip › Supplementary File S2 - Study Recruitment Clinical Information Checklist.pdf]

Medical Rehabilitation Sciences Department
Faculty of Applied Medical Sciences

UMM AL-QURA UNIVERSITY
Faculty of Applied Medical Sciences

قسم علوم التأهيل الطبي
كلية العلوم الطبية التطبيقية

STUDY RECRUITMENT AND CLINICAL INFORMATION CHECKLIST

Chronic Low Back Pain Study - Recruitment, Eligibility, Comorbidity, and Clinical Data Form

Purpose: Study-specific case-report/checklist form used to document recruitment eligibility and clinical information. This form is not a validated comorbidity index and does not replace the validated study questionnaires.

A. Administrative and Recruitment Information

**Participant study ID:**		**Screening date:**	____ / ____ / ______
**Consent obtained before study procedures:**	[ ] Yes [ ] No	**Eligibility status:**	[ ] Eligible [ ] Not eligible [ ] Pending

B. Eligibility Screening

Eligibility item	Yes	No	Notes / reason
Age 18 years or older	[ ]	[ ]	
Low back pain duration at least 12 weeks	[ ]	[ ]	
Participant reports a physician-diagnosed specific spinal pathology (fracture, infection, inflammatory disease, or malignancy)	[ ]	[ ]	
Spinal surgery within the previous 12 months	[ ]	[ ]	
Neurological, cognitive, or psychiatric condition of sufficient severity to impair accurate self-reporting or safe physical testing	[ ]	[ ]	
Able to understand study procedures and provide informed consent	[ ]	[ ]	

Eligibility decision rule: Eligible participants must meet the age and chronicity criteria and must not meet any exclusion criterion. A previous diagnosis of depression alone is not exclusionary unless its severity compromises accurate self-reporting or safe participation.

C. Demographic and Clinical Information

**Age (years):**		**Sex:**	[ ] Female [ ] Male
**Education level:**		**Employment status:**	[ ] Government [ ] Private [ ] Student [ ] Not working [ ] Other: ______
**Low back pain duration:**	[ ] 3 to <6 months [ ] >6 months	**Pain intensity (NRS 0-10):**	____ / 10
**Height (m):**		**Body mass (kg):**	
**BMI (kg/m2):**		**BMI category:**	[ ] <30 [ ] >=30
**Walking aid routinely used:**	[ ] No [ ] Yes: __________	**Other clinically relevant notes:**	

D. Comorbidity Checklist

Participant-report instruction: For each condition below, record whether the participant reports having previously received a clinical diagnosis. These diagnoses are not independently verified from medical records for this study. Obesity is classified separately by the research team from measured BMI.

Condition	Yes	No	Source / classification	Notes

Condition	Yes	No	Source / classification	Notes
Hypertension	[ ]	[ ]	Participant-reported previous diagnosis	
Diabetes mellitus	[ ]	[ ]	Participant-reported previous diagnosis	
Heart disease	[ ]	[ ]	Participant-reported previous diagnosis	
Osteoarthritis	[ ]	[ ]	Participant-reported previous diagnosis	
Asthma / COPD	[ ]	[ ]	Participant-reported previous diagnosis	
Depression	[ ]	[ ]	Participant-reported previous diagnosis	
Obesity (BMI >=30 kg/m2)	[ ]	[ ]	Research-team classification from measured height/body mass	

Comorbidity burden score: ____ / 7 Scoring: 1 point for each of the seven conditions present; 0 points if absent. Theoretical range 0-7. This is a study-specific unweighted count, not a validated weighted comorbidity index.

E. Study Measure Completion Log

Note: Validated questionnaires should be administered using their authorized study versions; this checklist records completion only and does not reproduce questionnaire content.

Measure	Completed	Not completed	Comments / reason
Fear-Avoidance Beliefs Questionnaire (FABQ-PA and FABQ-Work)	[ ]	[ ]	
Oswestry Disability Index (ODI)	[ ]	[ ]	
Pain Catastrophizing Scale (PCS)	[ ]	[ ]	
Pittsburgh Sleep Quality Index (PSQI)	[ ]	[ ]	

F. 6-Minute Walk Test (6MWT) - Safety and Performance Record

**Pre-test heart rate:**	_____ bpm	**Pre-test blood pressure:**	_____ / _____ mmHg
**Walking aid used during test:**	[ ] No [ ] Yes: __________	**Standard 30-m indoor course used:**	[ ] Yes [ ] No
**Standardized instructions/encouragement used:**	[ ] Yes [ ] No	**Rest or slowing required:**	[ ] No [ ] Yes
**Premature termination required:**	[ ] No [ ] Yes	**Reason if terminated:**	
**Safety symptoms during test:**	[ ] None [ ] Chest pain [ ] Severe dyspnea [ ] Dizziness [ ] Loss of balance [ ] Other: ______	**Post-test heart rate:**	_____ bpm
**Post-test blood pressure:**	_____ / _____ mmHg	**Total distance walked:**	________ m
**Adverse event requiring medical review:**	[ ] No [ ] Yes - describe below	**Additional notes:**	

G. Data Quality and Eligibility Confirmation

Eligibility criteria verified before inclusion	[ ] Yes [ ] No
Anthropometric data recorded	[ ] Yes [ ] No
Comorbidity checklist completed	[ ] Yes [ ] No
Questionnaire completion reviewed	[ ] Yes [ ] No
6MWT completed according to study procedure	[ ] Yes [ ] No [ ] Not completed

Assessor/researcher signature: ______________________________ Date: ____ / ____ / ______

Research use note

This checklist is designed to document recruitment and clinical information consistently with the study protocol and manuscript. It is study-specific and should be used alongside the approved informed-consent process and the validated instruments administered in the study.
