# Supplementary material for: Factors Associated with Functional Disability and Walking Capacity in Adults with Chronic Low Back Pain: A Cross-Sectional Study
Source: J Clin Med. 2026 Sep 29;15(19):7560. doi: 10.3390/jcm15197560 (PMC13666209; doi:10.3390/jcm15197560)
Supplement: Supplementary file 1 [file jcm-15-07560-s001.zip › Supplementary File S3 - Table S1-S5.pdf]

Supplementary Table S1. Distributional characteristics, internal consistency, and item-level response frequencies of the study measures

Panel A. Distributional characteristics and internal consistency

Measure	N	Theoretical range	Observed range	Mean ± SD	Median (IQR)	Skewness	Excess kurtosis	Cronbach's α
Pain intensity	339	0–10	0–10	5.95 ± 2.37	6 (4–8)	−0.155	−0.624	—
ODI	339	0–100	10–78	43.88 ± 14.22	44 (34–54)	0.073	−0.468	0.631
FABQ-PA	339	0–24	4–20	12.11 ± 4.84	12 (8–16)	0.016	−0.990	0.851
FABQ-Work	339	0–42	7–35	21.25 ± 8.34	21 (14–28)	0.055	−1.053	0.908

Panel B. Observed item-level response frequencies for scored FABQ items

FABQ subscale	Dataset	0	1	2	3	4	5	6
**FABQ-PA item 1**	**Q2**	**0**	**78**	**68**	**85**	**50**	**58**	**0**
**FABQ-PA item 2**	**Q3**	**0**	**66**	**54**	**65**	**54**	**100**	**0**
**FABQ-PA item 3**	**Q4**	**0**	**55**	**47**	**75**	**58**	**104**	**0**
**FABQ-PA item 4**	**Q5**	**0**	**103**	**51**	**75**	**46**	**64**	**0**
**FABQ-Work item 1**	**Q6**	**0**	**91**	**46**	**71**	**54**	**77**	**0**
**FABQ-Work item 2**	**Q7**	**0**	**113**	**52**	**74**	**39**	**61**	**0**
**FABQ-Work item 3**	**Q8**	**0**	**65**	**39**	**56**	**59**	**120**	**0**
**FABQ-Work item 4**	**Q9**	**0**	**33**	**33**	**72**	**72**	**129**	**0**
**FABQ-Work item 5**	**Q10**	**0**	**76**	**55**	**78**	**53**	**77**	**0**
**FABQ-Work item 6**	**Q11**	**0**	**125**	**43**	**65**	**40**	**66**	**0**
**FABQ-Work item 7**	**Q12**	**0**	**94**	**43**	**68**	**56**	**78**	**0**

Note. SD = standard deviation; IQR = interquartile range; ODI = Oswestry Disability Index; FABQ-PA = Fear-Avoidance Beliefs Questionnaire–Physical Activity subscale; FABQ-Work = Fear-Avoidance Beliefs Questionnaire–Work subscale. The administered FABQ retained the standard 0–6 response format, ranging from 0 ("completely disagree") to 6 ("completely agree"). In the present analytical sample, however, observed responses for all scored FABQ items ranged from 1 to 5; response categories 0 and 6 were available but were not observed. Each item-frequency row sums to N = 339. Cronbach's α is not applicable to the single-item pain-intensity measure. The 'Dataset' column reports the variable labels used in the study dataset. The FABQ-PA and FABQ-Work subscale totals were calculated from four and seven scored items, respectively.

Supplementary Table S2. Spearman rank correlations between comorbidity burden and study variables.

Variable	Spearman's ρ	*p*-value
Age	−0.022	0.682
Pain intensity	0.040	0.466
FABQ-PA	−0.072	0.184
FABQ-Work	−0.073	0.180
ODI	0.148	0.006
6MWT distance	−0.072	0.187

Abbreviations: FABQ-PA, Fear-Avoidance Beliefs Questionnaire–Physical Activity subscale; FABQ-Work, Fear-Avoidance Beliefs Questionnaire–Work subscale; ODI, Oswestry Disability Index; 6MWT, 6-Minute Walk Test. Note: Spearman rank correlations were conducted as a sensitivity analysis because comorbidity burden was represented as a count variable. N = 339.

Supplementary Table S3. Sensitivity analyses examining FABQ subscales separately in regression models of disability and walking capacity

Each model retained age, pain intensity, comorbidity burden, and sex. Models in Section C additionally included ODI. FABQ-PA and FABQ-Work were entered in separate models.

Functional disability (ODI)

Model / Predictor	B	SE	β	95% CI	*p*	R^2	Adj. R^2	F
Model A1: FABQ-PA						0.038	0.023	F(5,333)=2.606, p=0.025
Age	0.041	0.076	0.029	−0.109 to 0.191	0.590			
Pain intensity	0.531	0.346	0.088	−0.150 to 1.211	0.126			
FABQ-PA	−0.037	0.170	−0.012	−0.372 to 0.298	0.830			
Comorbidity burden	2.310	0.742	0.169	0.851 to 3.770	0.002			
Sex: male	−0.725	1.671	—	−4.011 to 2.561	0.665			
Model A2: FABQ-Work						0.038	0.024	F(5,333)=2.638, p=0.023
Age	0.041	0.076	0.029	−0.108 to 0.191	0.587			
Pain intensity	0.551	0.339	0.092	−0.117 to 1.218	0.106			
FABQ-Work	−0.043	0.097	−0.025	−0.233 to 0.147	0.656			
Comorbidity burden	2.293	0.742	0.168	0.833 to 3.753	0.002			
Sex: male	−0.693	1.666	—	−3.971 to 2.584	0.678			

Walking capacity (6MWT), without ODI

Model / Predictor	B	SE	β	95% CI	*p*	R^2	Adj. R^2	F
Model B1: FABQ-PA						0.050	0.036	F(5,333)=3.507, p=0.004
Age	0.033	0.556	0.003	−1.060 to 1.126	0.953			
Pain intensity	−5.211	2.526	−0.118	−10.179 to −0.243	0.040			
FABQ-PA	−2.750	1.243	−0.127	−5.196 to −0.304	0.028			
Comorbidity burden	−10.248	5.416	−0.102	−20.903 to 0.407	0.059			
Sex: male	1.235	12.198	—	−22.760 to 25.230	0.919			

Model B2: FABQ-Work						0.054	0.040	F(5,333)=3.825, p=0.002
Age	0.056	0.554	0.005	−1.035 to 1.146	0.920			
Pain intensity	−5.287	2.472	−0.120	−10.150 to −0.424	0.033			
FABQ-Work	−1.785	0.704	−0.142	−3.170 to −0.400	0.012			
Comorbidity burden	−10.502	5.408	−0.105	−21.140 to 0.136	0.053			
Sex: male	3.391	12.142	—	−20.495 to 27.276	0.780			

Walking capacity (6MWT), additionally adjusted for ODI

**Predictor**	**Model C1: FABQ-PA + ODI**					**Model C2: FABQ-Work + ODI**				
	**B**	**SE**	**β**	**95% CI**	***p***	**B**	**SE**	**β**	**95% CI**	***p***
Age	0.196	0.467	0.019	−0.722 to 1.114	0.674	0.221	0.464	0.021	−0.692 to 1.134	0.634
Pain intensity	−3.098	2.128	−0.070	−7.284 to 1.088	0.146	−3.086	2.078	−0.070	−7.174 to 1.003	0.139
FABQ-PA	−2.895	1.044	−0.134	−4.949 to −0.842	0.006	—	—	—	—	—
FABQ-Work	—	—	—	—	—	−1.957	0.590	−0.156	−3.118 to −0.797	0.001
Comorbidity burden	−1.049	4.613	−0.010	−10.124 to 8.025	0.820	−1.336	4.593	−0.013	−10.371 to 7.699	0.771
Sex: male	−1.652	10.244	—	−21.803 to 18.500	0.872	0.620	10.170	—	−19.387 to 20.626	0.951
ODI	−3.981	0.336	−0.542	−4.642 to −3.320	<0.001	−3.997	0.334	−0.544	−4.655 to −3.340	<0.001

Model C1: N = 339; $R^2 = 0.332$; adjusted $R^2 = 0.320$; $F(6,332) = 27.55$, $p < 0.001$.

Model C2: N = 339; $R^2 = 0.339$; adjusted $R^2 = 0.327$; $F(6,332) = 28.36$, $p < 0.001$.

Note. B = unstandardized regression coefficient; SE = standard error; β = standardized regression coefficient; CI = confidence interval; FABQ-PA = Fear-Avoidance Beliefs Questionnaire Physical Activity subscale; FABQ-Work = Fear-Avoidance Beliefs Questionnaire Work subscale; ODI = Oswestry Disability Index; 6MWT = 6-Minute Walk Test. Female was the reference category for sex. Standardized coefficients are not shown for the categorical sex variable.

Supplementary Table S4. Expanded sensitivity regression models for disability (ODI) and walking capacity (6MWT)

Exploratory multivariable linear regression models additionally adjusted for BMI, pain duration, pain catastrophizing (PCS), sleep quality (PSQI), and occupational status, while retaining the predictors from the primary models.

Disability (ODI) N = 339; $R^2 = 0.097$; adjusted $R^2 = 0.061$; $F(13, 325) = 2.70$, $p = 0.001$.

Predictor	B	SE	β	95% CI	*p*
Age	0.057	0.075	0.040	−0.091 to 0.205	0.451
BMI	−0.013	0.185	−0.004	−0.378 to 0.352	0.946
Pain intensity	0.536	0.343	0.089	−0.139 to 1.211	0.119
FABQ-PA	−0.132	0.275	−0.045	−0.674 to 0.410	0.633
FABQ-Work	−0.069	0.155	−0.040	−0.374 to 0.236	0.656
Comorbidity burden	2.345	0.733	0.172	0.904 to 3.787	0.002
PCS	0.035	0.078	0.024	−0.119 to 0.188	0.657
PSQI	0.283	0.227	0.067	−0.163 to 0.728	0.213
Pain duration >6 months	2.390	2.624	—	−2.772 to 7.553	0.363
Not working	1.176	2.063	—	−2.882 to 5.234	0.569
Private sector	−2.532	2.253	—	−6.964 to 1.901	0.262
Student	−7.753	2.246	—	−12.172 to −3.335	<0.001
Male	0.734	1.715	—	−2.641 to 4.109	0.669

Walking capacity (6MWT) N = 339; $R^2 = 0.311$; adjusted $R^2 = 0.283$; $F(13, 325) = 11.27$, $p < 0.001$.

Predictor	B	SE	β	95% CI	p
Age	−0.236	0.483	−0.023	−1.185 to 0.714	0.625
BMI (kg/m²)	−1.626	1.191	−0.064	−3.970 to 0.717	0.173
Pain intensity (0–10)	−4.773	2.204	−0.108	−9.108 to −0.438	0.031
FABQ–Physical Activity	1.664	1.769	0.077	−1.817 to 5.145	0.348
FABQ–Work	−1.030	0.995	−0.082	−2.988 to 0.927	0.301
Comorbidity burden	−11.113	4.706	−0.111	−20.371 to −1.855	0.019
Pain Catastrophizing Scale (PCS)	−0.073	0.501	−0.007	−1.059 to 0.913	0.885
PSQI (sleep quality)	−1.819	1.456	−0.059	−4.683 to 1.044	0.212
Pain duration: >6 months (reference: 3 to <6 months)	−4.064	16.855	—	−37.221 to 29.094	0.810
Occupation: not working (reference: Government sector)	−35.601	13.249	—	−61.666 to −9.537	0.008
Occupation: private sector (reference: Government sector)	43.573	14.471	—	15.105 to 72.041	0.003
Occupation: student (reference: Government sector)	122.663	14.424	—	94.286 to 151.039	<0.001
Sex: male (reference: female)	−16.288	11.018	—	−37.963 to 5.388	0.140

Note. B = unstandardized regression coefficient; SE = standard error; β = standardized regression coefficient; CI = confidence interval; ODI = Oswestry Disability Index; 6MWT = 6-Minute Walk Test; BMI = body mass index; FABQ = Fear-Avoidance Beliefs Questionnaire; PCS = Pain Catastrophizing Scale; PSQI = Pittsburgh Sleep Quality Index. JASP reports standardized coefficients only for continuous predictors; therefore, β is not shown for categorical predictors. Categorical coefficients are relative to the omitted reference category used in the regression model. These models were exploratory sensitivity analyses and did not replace the prespecified primary regression models. The FABQ-PA coefficient changed direction relative to the primary 6MWT model after broader covariate adjustment; sequential examination showed that the change occurred following inclusion of occupational status. The coefficient

remained nonsignificant and is therefore interpreted as model-dependent coefficient instability rather than evidence of a positive directional association.

Supplementary Table S5. *Sensitivity analysis of walking capacity using a modified ODI excluding the Walking and Standing items*

**Predictor**	**B**	**SE**	**β**	**95% CI**	***p***
Age	0.117	0.474	0.011	−0.815 to 1.048	0.806
Pain intensity	−3.087	2.161	−0.070	−7.338 to 1.164	0.154
Sex: male	3.047	10.450	—	−17.510 to 23.604	0.771
FABQ-PA	−0.452	1.715	−0.021	−3.826 to 2.921	0.792
FABQ-Work	−1.615	0.973	−0.129	−3.530 to 0.300	0.098
Comorbidity burden	−2.202	4.679	−0.022	−11.407 to 7.004	0.638
Modified ODI	**−3.591**	**0.321**	**−0.519**	**−4.222 to −2.960**	**<0.001**

Model: N = 339; $R^2 = 0.314$; adjusted $R^2 = 0.300$; $F(7,331) = 21.67$, $p < 0.001$.

Note. The modified ODI excluded the Walking and Standing items and was rescaled to a 0–100 score.
